# Vitamin D and depression in adults: A systematic review

**DOI:** 10.17305/bb.2025.12331

**Published:** 2025-04-30

**Authors:** Vlad Dionisie, Mihnea-Alexandru Găman, Cristina Anghele, Mihnea Costin Manea, Maria Gabriela Puiu, Iulia-Ioana Stanescu-Spinu, Octavian-Ilarian Baiu, Florian Antonescu, Mirela Manea, Adela Magdalena Ciobanu

**Affiliations:** 1 Department of Psychiatry and Psychology, “Carol Davila” University of Medicine and Pharmacy, Bucharest, Romania; 2 Department of Hematology, Faculty of Medicine, “Carol Davila” University of Medicine and Pharmacy, Bucharest, Romania; 3 Department of Hematology, Center for Clinical and Basic Research (CCBR Clinic), Bucharest, Romania; 4 Department of Cellular and Molecular Pathology, Stefan S. Nicolau Institute of Virology, Romanian Academy, Bucharest, Romania; 5 Department of Clinical Neurosciences, Discipline of Psychiatry, “Carol Davila” University of Medicine and Pharmacy, Bucharest, Romania; 6 Discipline of Physiology, Department – Dentistry III, Faculty of Dentistry, “Carol Davila” University of Medicine and Pharmacy, Bucharest, Romania; 7 “Prof. Dr. Alexandru Obregia” Clinical Hospital of Psychiatry, Bucharest, Romania; 8 Department of Neurology, “Carol Davila” University of Medicine and Pharmacy, Bucharest, Romania; 9 National Institute of Neurology and Neurovascular Diseases, Bucharest, Romania

**Keywords:** Vitamin D, cholecalciferol, ergocalciferol, calcitriol, deficiency, major depressive disorder, MDD, depression, mood disorders

## Abstract

Depression is one of the most prevalent psychiatric disorders and a leading cause of disability worldwide. Although the pathogenesis of depression remains far from fully understood, current research suggests a potential role for vitamin D due to its involvement in brain functioning. Moreover, vitamin D supplementation has shown promising results in the treatment of patients with depression. Therefore, the present study aimed to systematically review the available research investigating the association between vitamin D levels and the onset of depression. This systematic review was conducted according to the Preferred Reporting Items for Systematic Reviews and Meta-Analyses (PRISMA) guidelines, and the protocol was registered in the PROSPERO database (registration number: CRD42024515918). A search was performed across PubMed/Medline, SCOPUS, and Web of Science databases, yielding a total of 8,052 potentially eligible articles. After the removal of duplicates and ineligible records, and exclusion based on title and abstract screening, 297 original full-text articles were assessed according to the inclusion and exclusion criteria. Ultimately, 66 articles were included in this systematic review. Most of the included studies employed a cross-sectional design (*N* ═ 46). Overall, the data analyzed in this review indicate an association between depression and vitamin D serum levels, particularly in studies using cross-sectional designs. Only a few longitudinal studies demonstrated that lower vitamin D levels are associated with an increased risk of developing depressive symptoms or major depressive disorder, highlighting an important research gap. However, it remains to be established through future research whether acute or chronic vitamin D supplementation could have a protective effect against the development of depression.

## Introduction

**Figure 1.**
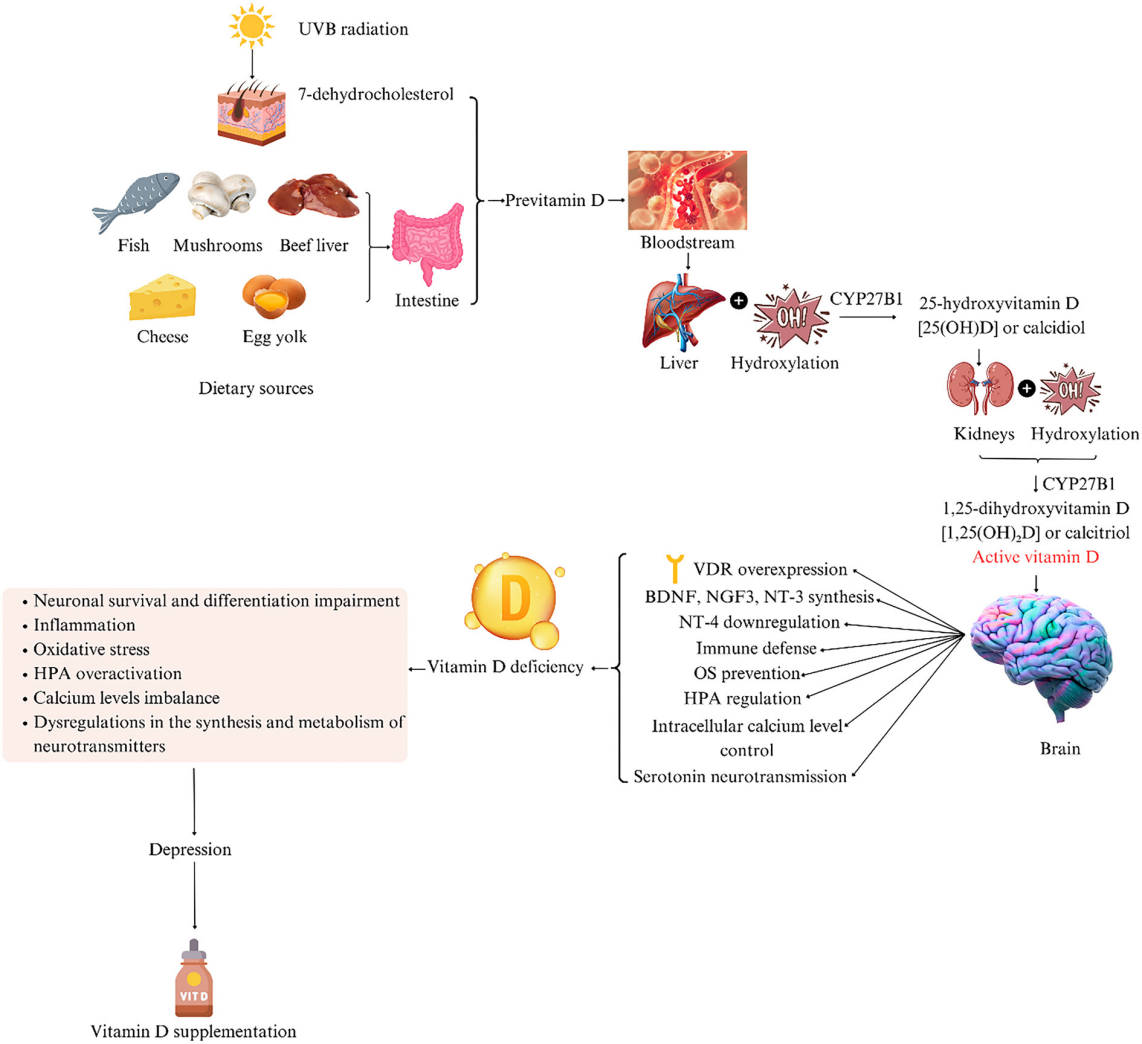
**Vitamin D—an overview of synthesis, metabolism, and roles in brain functioning and depression pathophysiology (figure created by the authors of the paper).** OS: Oxidative stress; HPA: Hypothalamic-pituitary-adrenal; BDNF: Brain-derived neurotrophic factor; VDR: Vitamin D receptor; NGF: Nerve growth factor; NT: Neurotrophin.

Depression is a globally prevalent illness that affects individuals of all ages and is frequently associated with both somatic and psychiatric comorbidities. According to the World Health Organization (WHO), 5% of all adults and 5.7% of adults over the age of 60 suffer from depression worldwide [1]. Depression is therefore linked to psychosocial disability [2] and reduced workplace productivity [3]. WHO estimates suggest that by 2030, depression will become the leading cause of disease burden globally [4]. Additionally, the COVID-19 Mental Disorder Collaborators report a significant increase in the prevalence and burden of major depressive disorder (MDD) as a consequence of the COVID-19 pandemic [5]. In light of this, researchers have made extensive efforts to identify the causes and mechanisms underlying the onset of depression. It is now understood that depression does not stem from a single cause, but rather results from a complex interplay of abnormalities, injuries, and deficits. While early evidence suggested that antidepressant treatments primarily addressed monoaminergic imbalance, more recent studies have shown that these drugs have broader effects, targeting multiple mechanisms involved in depression’s pathophysiology. Specifically, selective serotonin reuptake inhibitors (SSRIs) and serotonin-noradrenaline reuptake inhibitors (SNRIs) have demonstrated anti-inflammatory, antioxidant, pro-neurotrophic, and glucocorticoid-regulating properties [6–9]. Despite these findings, antidepressant medications commonly used in clinical practice demonstrate only small to moderate effectiveness [10]. One major factor appears to be non-adherence to prescribed treatment, which is notably higher in depression than in other medical conditions [11, 12]. However, studies also show that residual symptoms can persist even in patients who are adherent to treatment [13]. Therefore, a deeper understanding of the mechanisms involved in depression may contribute to the development of more effective and comprehensive therapeutic strategies. Vitamin D refers to a group of steroid molecules, of which vitamin D3 (cholecalciferol) and vitamin D2 (ergocalciferol) are the most important [14]. This fat-soluble vitamin is also known as the “sunshine vitamin,” as exposure to UVB radiation converts 7-dehydrocholesterol in the skin to pre-vitamin D [15]. Vitamin D is produced primarily in the skin or absorbed from dietary sources via the intestines [16], and both forms are subsequently converted to 25-hydroxyvitamin D in the liver. The active form, 1,25-dihydroxyvitamin D (calcitriol), is produced in the kidneys through a second hydroxylation step [17]. Calcitriol binds to the vitamin D receptor (VDR), which forms a complex with the retinoid X receptor and is translocated to the nucleus, where it activates transcription of target genes [14] (Figure 1). The role of vitamin D in normal brain function is suggested by the high expression of VDR in several brain regions, including the hypothalamus (involved in emotional regulation) [18] and the pons [19]. Furthermore, vitamin D is present in areas associated with the development of depression [20]. It also contributes to the synthesis of key neurotrophic factors essential for neuronal survival and differentiation, including the stimulation of brain-derived neurotrophic factor (BDNF), nerve growth factor 3, and neurotrophin-3, while downregulating neurotrophin-4 [20].

There are multiple hypotheses regarding the role of vitamin D in the pathophysiology of depression. One possible explanation involves inflammation. Vitamin D has been shown to play a role in immune defense by promoting the Th2 immune response and reducing the pro-inflammatory activity of Th1 cells [21]. However, its relationship with inflammation appears to be more complex. Recent findings suggest that inflammation can lead to vitamin D deficiency, while low levels of calcitriol may in turn promote inflammation [22]. In addition to inflammation, oxidative stress (OS) has been implicated in the development of MDD. Vitamin D supports the protective function of Nrf2 and contributes to normal mitochondrial function, thus regulating redox processes, which are essential for cell survival and proliferation. Vitamin D deficiency has been associated with increased oxidative damage, promotion of apoptosis, and the development of neurodegenerative diseases through the disruption of redox balance [23]. Administration of vitamin D has been shown to alleviate depression by inhibiting neuroinflammation and reducing OS levels [24]. Another mechanism by which vitamin D may exert antidepressant effects is through the regulation of neuronal calcium (Ca^2+^) levels, which become neurotoxic at high concentrations [25]. Vitamin D also helps maintain intracellular calcium homeostasis; its deficiency can disrupt the balance between glutamate, γ-aminobutyric acid (GABA), and calcium—a key factor in neuroexcitation and inhibition [26]. Experimental models have further demonstrated that vitamin D may enhance serotonin neurotransmission [27]. Vitamin D deficiency has been shown to impair the synthesis of 5-hydroxytryptamine (5-HT), a neurotransmitter considered a core factor in depression for over 50 years [20, 28]. In rats exposed to methamphetamine (a dopaminergic toxin), vitamin D treatment prevented the decline in serotonin and dopamine levels. Furthermore, developmental vitamin D deficiency resulted in alterations in dopamine and glutamate systems, while neonatal administration of vitamin D3 led to increased dopamine and noradrenaline levels in adult offspring. Calcitriol has also been found to influence the gene expression of tyrosine hydroxylase, an enzyme critical to catecholamine synthesis [29–31]. Vitamin D has also been implicated in neurotrophin regulation. It has been shown to induce the synthesis of BDNF, glial cell line-derived neurotrophic factor, and neurotrophin NT-3. Notably, vitamin D may activate the BDNF/TrkB signaling pathway [32]. Recent studies also report that cholecalciferol administration in rats chronically treated with corticosterone reduced depression-like behavior and modulated glucocorticoid receptors or lowered serum corticosterone levels [33, 34]. These findings suggest that vitamin D may influence hypothalamic-pituitary-adrenal (HPA) axis dysregulation, a key feature of depression. Consistent with these molecular findings, clinical studies have investigated the effect of vitamin D supplementation on depressive symptoms. An umbrella meta-analysis including 14 meta-analyses (10 randomized controlled trials and four cohort studies) found beneficial effects of vitamin D supplementation in patients with depression [35]. Similar conclusions were drawn by other meta-analyses [36, 37], and one meta-analysis of 29 studies reported that vitamin D supplementation reduced both the incidence and progression of depression [38]. However, the evidence remains inconsistent, as other studies have reported no improvement in depressive symptoms following vitamin D supplementation [39–41].

Given the ongoing uncertainty regarding the relationship between vitamin D levels and depression, despite numerous molecular and clinical studies, we conducted the present systematic review. Our objective was to examine the association between serum 25-hydroxyvitamin D levels (or categories thereof) and depressive symptoms or clinical depression in various adult populations. Additionally, this review aims to identify potential confounding factors influencing this relationship and to highlight gaps in the literature and future research directions. To our knowledge, this is the first systematic review to comprehensively address this research question.

## Materials and methods

The present systematic review was conducted in accordance with the Preferred Reporting Items for Systematic Reviews and Meta-Analyses (PRISMA) guidelines [42]. The review protocol was registered in PROSPERO (registration number: CRD42024515918).

### Search strategy

Three investigators (V.D., M.-A.G., and O.B.) independently conducted advanced searches in the PubMed/MEDLINE, Scopus, and Web of Science databases using specific keywords and keyword combinations. They retrieved relevant manuscripts published from the inception of each database through 30 April 2023. The keywords and combinations used in the systematic search were: (“Vitamin D” OR “Vitamin D Deficiency” OR Calciferol OR Ergocalciferol* OR Cholecalciferol* OR “Vitamin D2” OR “Vitamin D3” OR Calcifediol OR “25-Hydroxyvitamin D” OR “25-Hydroxycholecalciferol” OR Calcitriol OR “1,25-dihydroxyvitamin D” OR “1,25-dihydroxycholecalciferol”OR “25-hydroxyvitamin D [25(OH)D]” OR “25-hydroxyvitamin D2” OR “25(OH)D” OR “25(OH) vitamin D” OR Alphacalcidol OR alfacalcidol OR “25-Hydroxyvitamin D2” OR Dihydrotachysterol OR calcifediol) AND (“mood disorders” OR ”Depression” OR depress* OR “major depressive disorder*” OR MDD OR “depressive disorder*” OR “mood disorder*” OR “affective disorder*” OR “affective symptom*” OR dysthym* OR mood OR affective OR melancholia).

### Selection process, eligibility criteria and data extraction

The Rayyan tool was used to automatically identify duplicates [43]. The elimination of duplicates was performed manually by one of the investigators (V.D., M.-A.G., or O.B.). The three investigators independently screened titles and abstracts to identify manuscripts that potentially met the inclusion and exclusion criteria. Subsequently, the investigators retrieved and assessed the full text of eligible manuscripts and applied the inclusion and exclusion criteria. Any disagreement between the three investigators during title and abstract screening or full-text assessment of eligible manuscripts was resolved either by consensus or by consulting the senior researcher (A.M.C.).

The manuscripts were included in the systematic review if they met the inclusion criteria:

- Original studies (randomized controlled trials, cohort, case-control or cross-sectional studies) that have measured and reported the serum levels (or categories) of 25(OH)D levels.
- Original studies (randomized controlled trials, cohort, case-control, or cross-sectional studies) that reported the diagnosis of depression and/or the level of depressive symptoms. Diagnosis of depression was based on at least one of the following: a standardized psychiatric interview for Diagnostic and Statistical Manual of Mental Disorders (DSM) or International Classification of Disease (ICD) diagnosis criteria or a clinician made diagnosis of depression based on DSM or ICD criteria and/or a cut-off score of a validated and recognized rating scale. Levels of depression symptoms were measured using a validated and recognized scale.
- Original studies that examined and reported an independent association between 25(OH)D serum levels or categories of 25(OH)D levels and depression as an outcome of interest.
- Included studies controlled for relevant covariates/confounders and did not base their conclusions solely on univariate analysis/simple observations.
- Studies conducted on adult participants (≥18 years old).
- Articles were written in English, Romanian, French or Italian.

The following exclusion criteria were applied:

- Reviews, case reports, letter to the editors, abstracts presented at scientific meetings, book chapters, grey literature.
- Studies reporting research performed *in vitro* (cell cultures) or on animals.
- Studies conducted on specific populations (e.g., patients with diabetes mellitus, cardiovascular disease, etc; perinatal and postpartum women, postmenopausal, etc).
- Studies conducted on paediatric participants (age <18 years old).
- Articles written in other languages than English, Romanian, French or Italian.
- Study participants with another reported psychiatric diagnosis besides depression (bipolar disorder, schizophrenia, Alzheimer’s dementia, alcohol use disorder, etc.).
- Studies reporting no independent association between 25(OH)D serum levels or categories of 25(OH)D levels and depression as an outcome of interest.
- Interventional studies reporting the effect of vitamin D supplementation on depression.

The following data from the eligible studies were extracted by the reviewers in an Excel spreadsheet: first author surname, study location, design and population, method of depression assessment, method of 25(OH)D measurement, and main findings of the study.

Of note, due to the heterogeneity of the data, we were not able to perform a meta-analysis.

### Risk of bias and methodological quality appraisal

The risk of bias in the included manuscripts was assessed using the Mixed Methods Appraisal Tool (MMAT) [44], while the methodological quality of the included studies was evaluated using the Methodological Index for Non-Randomized Observational Studies (MINORS) [45]. The results of the quantitative assessment are reported in a supplementary file.

## Results

### Search results

**Figure 2.**
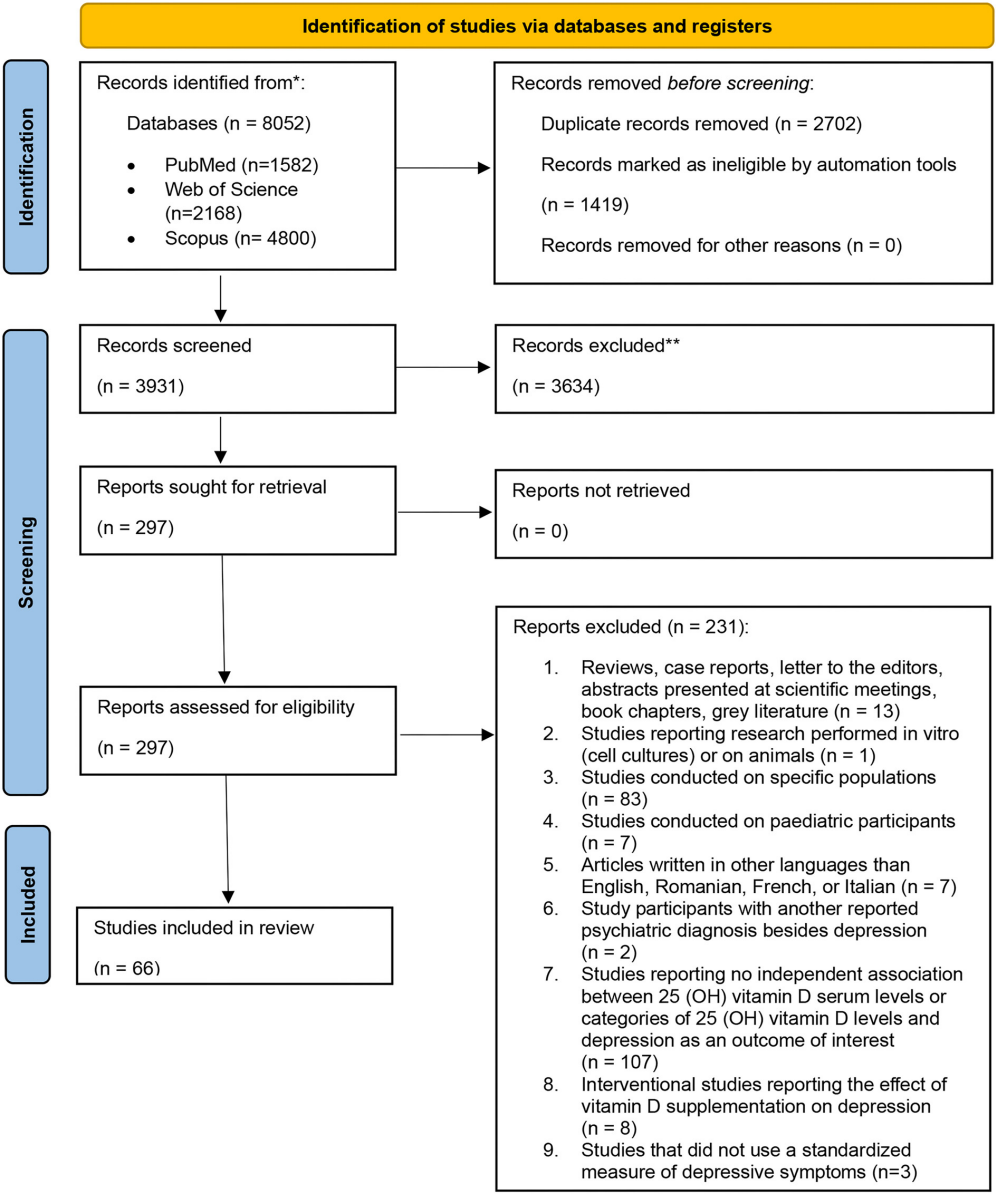
Prisma 2020 flow-chart diagram.

A total of 8052 papers were retrieved from the databases, with 2702 identified as duplicates and 1419 marked as ineligible by automation tools and eliminated. Of the remaining 3931, 3634 were excluded after the title and abstract screening step, and 231 were excluded after full-text screening based on inclusion and exclusion criteria. In the end, 66 articles were included in this systematic review (Figure 2) [46].

### Characteristics of the included studies

The studies included in this systematic review were published between 2008 and 2022, with a large number of articles published between 2020 and 2022 (*N* ═ 18). The studies were conducted on samples from 31 countries, with most of the research carried out in the United States of America and the Netherlands. Several methodological designs were employed: longitudinal only (*N* ═ 10), longitudinal and cross-sectional (*N* ═ 7), case-control (*N* ═ 3), and cross-sectional only (*N* ═ 46).

### Studies with longitudinal association analysis

A total of ten studies performed only a longitudinal analysis. Most studies (*N* ═ 6) used the Centre for Epidemiological Studies-Depression (CES-D) scale to assess the intensity of depressive symptoms, while other studies used the Inventory for Depressive Symptoms (IDS)-30SR. Milaneschi et al. (2019) assessed the risk of lifetime diagnosis of MDD and the association of 25(OH)D with depressive symptom severity in a cohort of 1700 cases of MDD. The lifetime diagnosis of MDD was established based on the DSM-IV criteria using the Composite Interview Diagnostic Instrument version 2.1 (WHO), administered at baseline and at two-year and four-year follow-ups. The researchers found that higher levels of 25(OH)D were associated with decreased odds of having a lifetime diagnosis of MDD (sex-adjusted OR ═ 0.78, 95% CI ═ 0.70–0.87, *P* ═ 1.4e-5). More specifically, it was observed that an increase of one standard deviation (i.e., 28.3 nmol/L) reduced the odds of a lifetime diagnosis of MDD by 22%. Also, higher levels of 25(OH)D were associated with decreased depressive symptoms as measured by the IDS-SR30 (beta ═ −1.5, SE ═ 0.25, *P* ═ 6.1e-10) [47]. Fashanu et al. (2019) examined the association between 25(OH)D levels and CES-D-11 scores. The researchers found no association between CES-D scores at the 20-year follow-up and baseline 25(OH)D categories (<20 ng/mL, 20–30 ng/mL), using the ≥30 ng/mL category as reference [48]. Briggs et al. (2019) found that community-dwelling individuals aged ≥50 years had increased odds of developing incident depression (CES-D-8 score ≥9) if they had insufficient (30–50 nmol/L) or deficient (<30 nmol/L) 25(OH)D levels compared to those with sufficient levels (>50 nmol/L) (OR ═ 1.04, 95% CI ═ 0.80–1.36; OR ═ 1.56, 95% CI ═ 1.07–2.26, respectively). The authors defined incident depression as a CES-D-8 score ≥9 at the two-year or four-year follow-up and excluded patients with depression at baseline (CES-D-20 score ≥16). The analysis excluded users of 25(OH)D supplements [49]. Elstgeest et al. (2018) assessed the relationship between changes in serum 25(OH)D and changes in CES-D-20 scores in an older cohort (individuals aged 65–88 years) and a younger cohort (individuals aged 55–65 years) over a period of 13 years and six years, respectively. The researchers observed that in the older cohort, neither the change in 25(OH)D levels as a continuous variable nor as tertiles was associated with a change in CES-D-20 score (25(OH)D as continuous variable: beta ═ −0.16, 95% CI ═ −0.70–0.37; 25(OH)D in tertiles: T1: beta ═ 1.63, 95% CI ═ −0.98–4.24; T2: beta ═ −0.28, 95% CI ═ −2.68–2.11; T3: reference, p for trend ═ 0.246). For the younger cohort, the authors divided participants into those with baseline serum 25(OH)D <58.6 nmol/L and those with >58.6 nmol/L. In participants with higher baseline 25(OH)D, no association was found between the change in serum 25(OH)D and change in CES-D-20 scores, regardless of variable type (i.e., continuous or tertiles). However, in participants with lower baseline 25(OH)D, each 10 nmol/L increase in 25(OH)D was associated with a 0.62-point decrease in CES-D-20 scores. Moreover, in the same subcohort, a negative change in 25(OH)D (i.e., tertile 1 ═ −54.0 to −2.7) was linked to an increase in CES-D-20 scores [50]. Collin et al. (2017) analyzed data from cancer-free patients with available CES-D-20 scores from the Supplémentation en Vitamines et Minéraux Antioxydants (SU.VI.MAX) cohort. The research group studied the risk of recurrent depressive symptoms, defined as a CES-D-20 score ≥16 at both baseline and 13-year follow-up. Individuals with 25(OH)D levels ≥10 ng/mL had a lower probability of recurrent depressive symptoms (PR ═ 0.48, 95% CI ═ 0.33–0.69, *P* < 0.001) compared to those with levels <10 ng/mL. However, when comparing participants with ≥20 vs <20 ng/mL or ≥30 vs ≤30 ng/mL, no significant differences in prevalence ratios were found (*P* ═ 0.17 and *P* ═ 0.39, respectively) [51]. van der Berg et al. (2021) evaluated the association between the course of depression or changes in IDS-SR30 scores and changes in 25(OH)D levels in a population (*N* ═ 232) of older adults (aged 60–93 years) with a DSM-IV diagnosis of depressive disorder (MDD and/or dysthymia or minor depression). The study was part of the Netherlands Study on Depression in Older Persons (NESDO). Although 25(OH)D levels decreased at the two-year follow-up, this was not associated with the course of depression (remission or non-remission). A reduction in IDS-SR30 score by one point was associated with a 0.22 nmol/L increase in 25(OH)D levels (estimate [SE] ═ 0.22 (0.11), effect size [95% CI] ═ 0.12 (0.00–0.24), *P* ═ 0.049), but this association was no longer significant after adjusting for frailty [52]. van der Berg et al. (2016) investigated the effect of 25(OH)D on the course of IDS-SR30 over time and depression status at two-year follow-up in a group of 367 individuals aged ≥60 years from the NESDO cohort. Neither 25(OH)D nor 1,25(OH)2D had a significant effect on depression status at follow-up. Furthermore, the authors found no effect of either vitamin D marker on total IDS-SR30 scores or its subscales (mood, motivational, and somatic) over time. Similar results were found when 25(OH)D deficiency was defined as levels <25 nmol/L [53].

William et al. (2015) investigated the risk of incident depression over a four-year follow-up period according to 25(OH)D categories (<20 ng/mL, 20–30 ng/mL, ≥30 ng/mL) in a cohort of older persons (aged 70–79 at the time of enrollment) (The Health ABC Study). Participants’ depressive symptoms were assessed at baseline and at one-, two-, three-, and four-year follow-ups, and 25(OH)D levels were measured at the one-year follow-up. The researchers found that participants with 25(OH)D levels <20 ng/mL and between 20–30 ng/mL had a 1.65 and 1.31 times higher risk, respectively, of developing incident depression compared to participants with 25(OH)D levels ≥30 ng/mL (*P* for trend <0.001) [54]. Wainberg et al. (2021) evaluated the risk of incident MDD over a five-year period in a large cohort of individuals (*N* ═ 433,890) aged 40–69 at the time of recruitment, from the UK Biobank. It was demonstrated that low baseline 25(OH)D levels were not associated with an increased risk of incident MDD over the five-year period [55]. Another longitudinal study conducted by Kerr et al. (2015) [56] investigated the relationship between changes in 25(OH)D levels between week one and week five and clinically significant depressive symptoms (i.e., CES-D score ≥16) in a group of undergraduate women recruited during the autumn, winter, or spring terms. The authors found that decreases in 25(OH)D levels between the two measurement points were associated with an increased probability of clinically significant depressive symptoms [56]. The main findings of this section are summarized in Table 1.

**Table 1:** Main findings of the studies with longitudinal association analysis

**Author and year**	**Study location**	**Study design**	**Study population**	**Depression assessment**	**Main findings**
Milaneschi et al. (2019) [47]	The Netherlands	NESDA, prospective cohort, two- and four-year follow-up	Patients with dx of lifetime MDD and healthy controls	DSM-IV, IDS-SR30	*↑*25(OH)D were associated with *↓*odds of having a lifetime diagnosis of MDD (sex-adjusted OR ═ 0.78, 95% CI ═ 0.70–0.87, *P* ═ 1.4e-5)
Fashanu et al. (2019) [48]	USA	ARIC-NCS, prospective cohort, 20-year follow-up	Participants aged 45–65 years, dementia-free at 25(OH)D measurement	CES-D-11-item	No association between CES-D scores and 25(OH)D category (bet-coefficients (95% CI): ≥30 (sufficient) ═ 0 (reference); 20–30 (intermediate) ═ −0.07 (−0.25, 0.12); <20 (Deficient) ═ −0.09 (−0.31, 0.13); *P* for trend ═ 0.4)
Briggs et al. (2019) [49]	Ireland	TILDA, prospective, cohort, two- and four-year follow-up	Community dwelling older individuals aged ≥50 years; patients with CES-D-20-item score ≥16 at baseline were excluded	CES-D-8-item, score ≥9 ═ incident depression	Participants with insufficient (30–50 nmol/L) or deficient (<30 nmol/L) 25(OH)D had *↑*odds of having incident depression in comparison with those with sufficient 25(OH)D (>50 nmol/L) (OR ═ 1.04, 95% CI ═ 0.80–1.36 and OR ═ 1.56, 95% CI ═ 1.07–2.26)
Elstgeest et al. (2018) [50]	The Netherlands	LASA, prospective cohort, 13-year follow-up for older cohort and six-year follow-up for younger cohort	Older adults, older cohort (55–85 years) and younger cohort (55–65 years)	CES-D-20-item	Older cohort: change in 25(OH)D level (continuous variable) and change in 25(OH)D (tertiles) were not associated with change in CES-D-20 score (25(OH)D continuous: beta ═ −0.16, 95% CI ═ −0.70 to 0.37; 25(OH)D tertiles: T1: beta ═ 1.63, 95% CI ═ −0.98 to 4.24, T2: beta ═ −0.28, 95% CI ═ −2.68 to 2.11, T3: reference, *P* for trend ═ 0.246). Younger cohort (25(OH)D>58.6 nmol/L): no association between change in 25(OH)D (continuous or tertiles) and change in CES-D-20 score (25(OH)D continuous: beta ═ 0.27, 95% CI ═ −0.22 to 0.76; tertiles: T1: beta ═ −0.85, 95% CI ═ −3.00 and 1.30, T2: beta ═ −0.92, 95% CI ═ −3.16 to 1.31, T3: reference. Younger cohort (25(OH)D < 58.6 nmol/L: change in CES-D score was associated with change in 25(OH)D (continuous) (beta ═ −0.61, 95% CI ═ −1.17, −0.07, *P* < 0.05) and 25(OH)D (tertiles) (T1: beta ═ 3.07, 95% CI ═ 0.73–5.40, *P* < 0.05; T2: beta ═ 1.67, 95% CI ═ −0.35 to 3.69, *P* > 0.05, T3: reference; *P* for trend ═ 0.008) T1 ═ −54.0 to −2.7, T2 ═ −2.7 to 11.8, T3 ═ 11.9 to –81.3 nmol/L
Collin et al. (2017) [51]	France	SU.VI.MAX 2, prospective cohort, 13-year follow-up	General population	CES-D-20 item, score ≥16 at baseline and follow-up ═ recurrent depressive symptoms	Individuals with 25(OH)D≥10 ng/mL had a *↓* probability of having recurrent depressive symptoms vs those with 25(OH)D < 10 ng/mL: PR ═ 0.48, 95% CI ═ 0.33–0.69, *P* < 0.001 ≥20 vs <20 ng/mL: PR ═ 0.79, 95% CI ═ 0.57–1.11, *P* ═ 0.17 ≥30 vs <30 ng/mL: PR ═ 0.82, 95% CI ═ 0.52–1.29, *P* ═ 0.39
van der Berg et al. (2021) [52]	The Netherlands	NESDO, prospective cohort, two-year follow-up	Older adults (60–93 years)	DSM-IV (MDD and/or dysthymia or minor depression), IDS-SR30	Change in 25(OH)D was not associated with depression course at follow-up (estimate(std. error) ═ −4.61(2.60), effect size ═ −0.20, 95% CI ═ −0.41–0.02, *P* ═ 0.077) *↑* in 25(OH)D was associated with a *↓* in IDS-SR30 (estimate[std. error] ═ 0.22(0.11), effect size ═ 0.12, 95% CI ═ 0.00–0.24, *P* ═ 0.049) Association between 25(OH)D with IDS-SR30 and frailty simultaneously, showed no effect on change in IDS-30SR score (estimate[std. error] ═ 0.18 (0.13), effect size ═ 0.10, 95% CI ═ −0.04 to 0.23, *P* ═ 0.157)
van der Berg et al. (2016) [53]	The Netherlands	NESDO, prospective cohort, two-year follow-up, severity of depressive symptoms assessed every 6 months	Older adults patients (60–93 years) from mental health institutions or GPs; patients with suspected dementia or MMSE<18/30p excluded	DSM-IV-TR (MDD, dysthymia, minor depression), IDS-SR30	No effect of 25(OH)D, 1,25(OH)D2 or 25(OH)D deficiency (<25 nmol/L) on the course of IDS-SR30 scores over time (B[s.e.] ═ 0.11(0.17), *P* ═ 0.503; B[s.e.] ═ 0.06(0.17), *P* ═ 0.705; B[s.e.] ═ −0.64(0.58), *P* ═ 0.265, respectively)
William et al. (2015) [54]	USA	Health ABC, prospective cohort, two-, three- and four-year follow-up	Older adults (70–79 years)	CES-D-10, GSD-15, taking antidepressant medication	Having a lower level of 25(OH)D was associated with an increased risk of having incident depression over four years (<20 ng/mL: HR ═ 1.65, 95% CI ═ 1.23–2.22; 20–30 ng/mL: HR ═ 1.31, 95% CI ═ 0.99–1.74; ≥30 ng/mL: reference; *P* for trend <0.001)
Wainberg et al. (2021) [55]	UK	UK Biobank, prospective cohort, five-year follow-up	Adults aged 40–69 years	ICD-10 (F32 diagnosis)	Low baseline levels of 25(OH)D were not associated with higher depressive episode incidence over five years
Kerr et al. (2015) [56]	USA	Prospective, five weekly follow-ups	Undergraduate women aged 18–25 years	CES-D-20, score ≥16 ═ clinically significant depressive symptoms. LC-MS/MS	The change in levels of 25(OH)D between weeks one and five was negatively associated with clinically significant depressive symptoms across five weekly assessments (Est [SE] ═ −0.02 (0.01), beta ═ −0.20)

NESDO: Netherlands Study on Depression in Older Persons; DSM: Diagnostic and Statistical Manual of Mental Disorders; IDS: Inventory for Depressive Symptoms; LC-MS-MS: Liquid chromatography-tandem mass spectrometry; CES: Centre for Epidemiological Studies-Depression; SU.VI.MAX: Supplémentation en Vitamines et Minéraux Antioxydants; ICD: International Classification of Disease.

### Studies with cross-sectional and longitudinal association analyses

Jovanova et al. (2017) investigated the cross-sectional and longitudinal relationship between 25(OH)D levels and the intensity of depressive symptoms or incident MDD in a cohort of residents of a district in Rotterdam aged ≥55 years. In the cross-sectional analysis, the authors reported a significant negative association between CES-D score and 25(OH)D serum levels. Moreover, participants with serum levels ≤37.5 nmol/L, ≤50 nmol/L, and ≤75 nmol/L had higher CES-D scores compared with those with vitamin D serum levels >37.5 nmol/L, >50 nmol/L, and >75 nmol/L, respectively. The longitudinal analysis revealed different results. 25(OH)D serum levels were not associated with depressive symptoms at the first or second follow-up, nor with the change in CES-D score between follow-ups. Additionally, the authors showed that serum levels of 25(OH)D did not predict the long-term risk of incident MDD [57].

The study by Almeida et al. (2015) analyzed the association between baseline 25(OH)D serum levels and either a current or past diagnosis of depression, as well as the risk of incident depression during a six-year follow-up. The authors showed that while a 25(OH)D serum level <30 nmol/L was associated with 2.7 times higher odds of having current depression, a level <50 nmol/L (<30 nmol/L or 30–49 nmol/L) was not associated with increased odds of past depression. Moreover, although 81 out of 2740 men had significant symptoms of depression during the follow-up period, the authors did not find an increased risk of incident depression associated with lower 25(OH)D serum levels [58].

Sahasrabudhe et al. (2020) conducted a study on the association between CES-D scores and 25(OH)D serum levels in a cohort of 1499 Puerto Rican adults from the Boston area. The cross-sectional analysis showed that participants with deficient or insufficient plasma levels of 25(OH)D (<12 ng/mL and 12–20 ng/mL, respectively) did not have a higher risk of greater intensity of depressive symptoms compared with those with plasma 25(OH)D levels in the sufficient category (≥20 ng/mL). Longitudinal analysis revealed similar results. Participants with insufficient or deficient 25(OH)D plasma levels at baseline did not have a significantly higher risk of worse CES-D score change at the five-year follow-up [59]. Milaneschi et al. (2010) investigated the relationship between 25(OH)D and depressive symptoms (measured using the CES-D scale) in a sample of older adults (≥65 years) from the InCHIANTI longitudinal study in Italy. The researchers reported that, for both men and women, lower levels of 25(OH)D (i.e., in tertiles 1 and 2) were not cross-sectionally associated with higher CES-D scores compared with higher levels of 25(OH)D (i.e., tertile 3). When 25(OH)D levels were dichotomized using a cut-off threshold of 50 nmol/L, similar results were obtained. However, at the three-year follow-up, both men and women with lower levels of 25(OH)D at baseline (i.e., in tertile 2: 31.7–53.9 nmol/L) experienced increases of 1.91 and 2.20, respectively, in CES-D score compared with those with higher 25(OH)D levels (i.e., tertile 3: ≥53.9 nmol/L). At the six-year follow-up, only women with lower 25(OH)D levels at baseline (i.e., <50 nmol/L) had an increase of 2.19 in CES-D score. Regarding the risk of depressed mood, only women with lower 25(OH)D levels at baseline (i.e., in tertile 1: <31.7 nmol/L or <50 nmol/L) had a 2.56-fold and 1.97-fold increased risk of higher CES-D score, respectively [60].

De Koning et al. (2018) analyzed data from two cohorts of the LASA study, involving predominantly older participants (55–85 years old for the older cohort and 55–65 years old for the younger cohort). Participants were followed up at three-year and six-year intervals, and depressive symptoms were assessed using the CES-D scale. For both cohorts, the authors reported that a level of 25(OH)D in the lower categories (i.e., <30 nmol/L, 30–50 nmol/L, and 50–75 nmol/L) was not cross-sectionally associated with increased intensity of depressive symptoms when compared with 25(OH)D in the highest category (>75 nmol/L). In the longitudinal analysis of the older cohort, the authors showed that women with lower 25(OH)D levels were associated with greater depressive symptoms at three-year and six-year follow-up, but this association was not observed in men. For the younger cohort, there was no significant longitudinal relationship between lower categories of 25(OH)D and CES-D scores [61]. Chan et al. (2011) studied the possible association between 25(OH)D and depression at baseline and at four-year follow-up in a cohort of older men (*N* ═ 883) without cognitive impairment (i.e., participants with a Community Screening Instrument for Dementia score ≤28.4 were excluded from the analysis). Individuals with 25(OH)D plasma levels in quartile 4 (≥92 nmol/L) had 54% decreased odds of having depression (defined as a geriatric depression scale (GDS)-15 score ≥8) in comparison with men with 25(OH)D in quartile 1 (≤63 nmol/L). Similar results were reported when conventional cut-offs of 25(OH)D were employed. At four-year follow-up, 25(OH)D level at baseline was not associated with incident depression [62].

Toffanello et al. (2014) conducted an Italian population-based cohort study of individuals aged 65 years and older, investigating the association between GDS scores and vitamin D categories. The authors found that only female participants with deficient 25(OH)D (<50 nmol/L) had higher GDS scores than those with better levels of 25(OH)D (i.e., >50 and ≤75 nmol/L or >75 nmol/L). Moreover, at follow-up (4.4 years), neither men nor women with deficient or insufficient (>50 and ≤75 nmol/L) 25(OH)D levels had a significantly increased risk of developing depressive symptoms compared to those with sufficient levels (>75 nmol/L) [63]. The main findings of this section are summarized in Table 2.

**Table 2:** Main findings of the studies with cross-sectional and longitudinal association analyses

**Author and year**	**Study location**	**Study design**	**Study population**	**Depression assessment** **Method of 25(OH)D measurement**	**Main findings**
Jovanova et al. (2017) [57]	The Netherlands	Rotterdam study; cross-sectional and prospective; baseline (1997–1999), first follow-up (2002-2004), second follow-up (2009–2011), 10 (SD ═ 3.5)-year total follow-up	Residents of a district in Rotterdam aged ≥55 years	CES-D-20, DSM-IV (SCAN), GP medical records of MDD episodes ECLIA	Cross-sectional analysis: *↓*25(OH)D was associated with *↑*CES-D score (beta ═ −0.27, 95% CI ═ −0.51; −0.04, *P* ═ 0.023); 25(OH)D ≤ 37.5 nmol/L was associated with *↑*CES-D score (beta ═ 0.48, 95% CI ═ −0.01–0.95, *P* ═ 0.046 ) compared with 25(OH)D > 37.5 nmol/L; 25(OH)D ≤ 75 nmol/L was associated with *↑*CES-D score (beta ═ 0.61, 95% CI ═ −0.02 to 1.20, *P* ═ 0.045) compared with 25(OH)D > 75 nmol/L; lower quartiles (<28.57 nmol/L, 28.58–43.81 nmol/L, 43.82–63.21 nmol/L) were associated with *↑*CES-D score compared with reference quartile (>63.21 nmol/L) Longitudinal analysis: 25(OH)D was not associated with CES-D score at first or second follow-up or with change in CES-D score between follow-ups; 25(OH)D did not predict the risk of incident MDD (HR ═ 0.95, 95% CI ═ 0.86–1.05, *P* ═ 0.61); similar results regarding CES-D scores and risk of incident MDD were reported for 25(OH)D cut-offs (≤37.5 nmol/L vs >37.4 nmol/L, ≤50 nmol/L vs >50 nmol/L, ≤75 nmol/L vs >70 nmol/L) and quartiles (<28.57 nmol/L, 28.58–43.81 nmol/L, 43.82–63.21 nmol/L vs >63.21 nmol/L)
Almeida et al. (2015) [58]	Australia	HIMS; retrospective, cross-sectional and prospective; baseline (2001–2004), follow-up (2008), 6 (SD ═ 2.2, range ═ 0.1–10.9 years) years of follow-up	Older men (aged 71–88 years,) living in Western Australia	Depression group at the time of assessment: Past depression ═ ICD-9 or ICD-10 based recorded diagnosis of depression, having been told for the first time by a doctor that he has depression or use of an antidepressant at the time of blood sampling; Current depression ═ GDS-15 ≥ 7 Incident depression group: PHQ-9 ≥ 10 at follow-up or ICD-9 or ICD-10 based recorded diagnosis of depression between baseline and follow-up CLIA	Cross-sectional analysis: 25(OH)D <30 nmol/L is associated with current depression (OR ═ 2.70, 95% CI ═ 1.39–5.25, *P* < 0.05) compared with 25(OH)D ≥ 50 nmol/L; 25(OH) < 30 nmol/L nor 25(OH) ═ 30–49 nmol/L were not associated with past depression (OR ═ 1.15, 95% CI ═ 0.56, 2.34, *P* > 0.05; OR ═ 1.10, 95% CI ═ 0.78–1.57, *P* > 0.05) compared to 25(OH)D ≥ 50 nmol/L Longitudinal analysis: Men with 25(OH)D <50 nmol/L had no *↑*risk of incident depression (HR ═ 1.03, 95% CI ═ 0.59–1.79 compared with those with 25(OH)D ≥50 nmol/L; similar results for men with 25(OH)D ═ 30–49 nmol/L and <30 nmol/L compared with those with 25(OH)D ≥50 nmol/L (HR ═ 0.97, 95% CI ═ 0.53–1.78 and HR ═ 1.38, 95% CI ═ 0.43–4.45, respectively)
Sahasrabudhe et al. (2020) [59]	USA	BPRHS; cross-sectional and prospective, two- and five-year follow-up	Self-identified Puerto Rican adults from Boston area, aged 45–75 years, participant with low MMSE excluded	CES-D-20 RIA	Cross-sectional analysis: Deficient or insufficient 25(OH)D categories were not associated with a higher CES-D score compared with sufficient category (beta ═ −0.85, 95% CI ═ −2.80 to 1.10) and beta ═ 0.50, 95% CI ═ −1.00 to 2.00, respectively; *P* for trend ═ 0.59) Longitudinal analysis: Baseline 25(OH)D deficient or insufficient categories were not associated with change in CES-D score over five years compared with sufficient category (beta ═ −0.41, 95% CI ═ −1.95 to 1.13 and beta ═ 0.82, 95% CI ═ −0.37 to 2.02, respectively; *P* for trend ═ 0.93). Similar results were reported for participants with complete data on CES-D score at all three assessments Insufficient: <12 ng/mL; Deficient: 12–20 ng/mL; Sufficient: ≥20 ng/mL
Milaneschi et al. (2010) [60]	Italy	InCHIANTI; cross-sectional and prospective, three-year (2001–2003) and six-year (2004–2006) follow-up	Older population, aged ≥65 years	CES-D-20, score ≥16 ═ depressed mood RIA	Cross-sectional analysis: Neither men or women with 25(OH)D in tertiles 1 or 2 were associated with increased CES-D scores compared with those with 25(OH)D in tertile 3 (for men: beta [SE] ═ 0.04 (1.0), *P* ═ 0.97 and beta [SE] ═ –1.02 (0.7), *P* ═ 0.14, respectively; for women: beta [SE] ═ −0.53 (1.1), *P* ═ 0.63 and beta [SE] ═ 0.65 (1.1), *P* ═ 0.55, respectively). Similar results were reported for 25(OH)D <50 nmol/L vs ≥50 nmol/L Longitudinal analysis: At three-year follow-up, 25(OH)D in tertile 2 in men and women were associated with increased CES-D score compared to 25(OH)D in tertile 3 (beta [SE] ═ 1.91 (0.9), *P* ═ 0.03 and beta [SE] ═ 2.20 (1.0), *P* ═ 0.03, respectively). Similar results were reported for 25(OH)D <50 nmol/L vs ≥50 nmol/L (beta [SE] ═ 1.91 (0.8), *P* ═ 0.01 and beta [SE] ═ 2.05 (0.9), *P* ═ 0.02, respectively). At six-year follow-up, 25(OH)D <50 nmol/L in women were associated with increased CES-D scores compared to 25(OH)D ≥50 nmol/L (beta[SE] ═ 2.19 (1.1), *P* ═ 0.04). For women, 25(OH)D at baseline in tertile 1 were associated with an 2.56-fold increased risk of depressed mood compared with 25(OH)D in tertile 3 (HR ═ 2.56, 95% CI ═ 1.41–4.64, *P* ═ 0.002) and 25(OH)D <50 nmol/L were associated with 1.97-fold increased risk of depressed mood compared with 25(OH)D ≥50 nmol/L (HR ═ 1.97, 95% CI ═ 1.22–3.17, P ═ 0.005). Tertile 1: <31.7 nmol/L; tertile 2: 31.7–53.9 nmol/L; tertile 3: ≥53.9 nmol/L
de Koning et al. (2018) [61]	The Netherlands	LASA, cross-sectional and prospective three-year and six-year follow-up (1998–1999 and 2001–2002 for older cohort; 2005–2006 and 2008–2009 for younger cohort)	Predominantly older adults (55–85 years old for older cohort and 55–65 years old for younger cohort)	CES-D-20. Younger cohort: CPBA; Older cohort: RIA	Cross-sectional analysis: In the younger-old cohort and older cohort, there was not a significant relationship between 25(OH)D and CES-D score (F (df1, df2) ═ 2.16 (3, 1238), *P* ═ 0.091 and F (df1, df2) ═ 1.35 (3, 710), *P* ═ 0.26, respectively) Longitudinal analysis: Older cohort: For women, 25(OH)D <30 nmol/L, 30–50 nmol/L and 50–75 nmol/L at baseline were associated with greater depressive symptoms at three-year and six-year follow-up compared with 25(OH)D >75 nmol/L (ratio [SE] ═ 1.23 (1.10), ratio [SE] ═ 1.17 (1.08) and ratio [SE] ═ 1.24 [1.08], respectively; F (df1, df2) ═ 2.60 (3, 553), *P* ═ 0.05). No significant associations were reported in men (F (df1, df2) ═ 0.12 (3, 464), *P* ═ 0.95). Younger cohort: No significant relationship between 25(OH)D and depressive symptoms was reported (F (df1, df2) ═ 1.91 (3, 670), *P* ═ 0.13).
Chan et al. (2011) [62]	China	OS study, cross-sectional and longitudinal, four-year follow-up	Older men aged ≥65years old without cognitive impairment	GDS-15, score ≥8 ═ depression RIA	Cross-sectional analysis: Men with baseline 25(OH)D in Q4 had lower odds of having depression compared to Q1 (OR ═ 0.46, 95% CI ═ 0.22–0.98, *P* for trend ═ 0.004). A similar trend was observed for 25(OH)D conventional categories (*P* for trend ═ 0.021). Longitudinal analysis: There was no association between baseline 25(OH)D and incident depression at the four-year follow-up (by quartiles: *P* for trend ═ 0.816; by conventional categories: *P* for trend ═ 0.619) Q1: ≤63 nmol/L, Q2: 64–76 nmol/L, Q3: 77–91 nmol/L, Q4: ≥92 nmol/L Conventional categories: ≥100 nmol/L, 75–99 nmol/L, 50–74 nmol/L, <50 nmol/L
Toffanello et al. (2014) [63]	Italy	Pro. V.A., cross-sectional and longitudinal 4.4 year follow-up	Older adults aged ≥65years old from the Northeast of Italy	GDS-30 RIA	Cross-sectional analysis: Only in female participants, there was observed a significant decreasing linear trend in the GDS scores across 25(OH)D categories (≤50 nmol/L, >50–≤75 nmol/L, >75 nmol/L) (*P* ═ 0.02). This trend was not observed in male participants Longitudinal analysis: There was no association between baseline 25(OH)D category and incident depression at the follow-up

CES-D: Centre for Epidemiological Studies-Depression; ICD: International Classification of Disease; GDS: Geriatric Depression Scale; DSM: Diagnostic and Statistical Manual of Mental Disorders; CPBA: Competitive protein binding assay; OS: Oxidative stress; RIA: Radioimmunoassay.

### Case-control studies

Sotoudeh et al. (2020) conducted a case-control study on 330 participants (110 MDD patients and 220 controls matched based on sex, age, and residential area) in Iran. MDD was diagnosed based on DSM-IV criteria, and healthy participants were selected based on their Beck Depression Inventory (BDI)-II scores. The researchers observed that MDD patients had decreased odds of having elevated 25(OH)D levels compared to healthy participants. Thus, it was concluded that lower 25(OH)D levels were associated with having MDD [64]. Milaneschi et al. (2014) studied the odds of having decreased levels of 25(OH)D in individuals with remitted or current MDD compared to healthy individuals. The researchers used the Composite Interview Diagnostic Instrument (based on DSM-IV criteria) to establish the diagnosis of a remitted or current MDD episode and the IDS to measure depression severity. It was observed that patients with a current MDD episode had an 80% and 117% increased odds of having insufficient or deficient 25(OH)D levels, respectively, compared to healthy controls. Similar results (i.e., a 68% increase in the odds of having insufficient 25(OH)D) were obtained for patients with a remitted MDD episode compared to healthy controls. Moreover, low levels of 25(OH)D were associated with increased severity of the current MDD episode [65].

Grudet et al. (2022) conducted a study on difficult-to-treat depressive patients. The MDD group comprised patients with MDD single episode, MDD recurrent episode, chronic MDD, or dysthymia, as defined by DSM-IV-TR criteria. The authors reported that patients with an insufficient treatment response had decreased odds of having increased 25(OH)D levels. In other words, every increase in 25(OH)D levels by 10 nmol/L was associated with a 17% decrease in the odds of having difficult-to-treat depression [66]. The main findings of this section are summarized in Table 3.

**Table 3:** Main findings of the case-control studies

**Author and year**	**Study location**	**Study design**	**Study population**	**Depression assessment** **Method of 25(OH)D measurement**	**Main findings**
Sotoudeh et al. (2020) [64]	Iran	Case-control	330 participants (110 MDD, 220 controls) aged 18–65 years Matched based on sex, age, and residential area	DSM-IV (MDD group), BDI-II (control group) EI	MDD patients were associated with *↓*odds of having *↑*25(OH)D levels compared with healthy participants (OR ═ 0.93, 95% CI ═ 0.87,0.99, *P* < 0.05)
Milaneschi et al. (2014) [65]	The Netherlands	NESDA, case-control	2386 participants (494 controls, 790 with remitted MDD, 1102 with current MDD) aged 18–65 years Controls ═ no symptoms of anxiety or depressive disorders	DSM-IV (CIDI) for remitted or current MDD DSM-IV (CIDI) for any lifetime depressive/anxiety disorder and IDS score <14 for controls ID-XLC-MS/ MS	Patients with current MDD episode had *↑*odds of having insufficient or deficient 25(OH)D levels compared with healthy controls levels (OR ═ 1.80, 95% CI ═ 1.29–2.51, *P* ═ 0.001 and OR ═ 2.17, 95% CI ═ 1.24–3.80, *P* ═ 0.01, respectively); patients with remitted MDD episode had *↑*odds of having insufficient 25(OH)D levels compared with healthy controls (OR ═ 1.68, 95% CI ═ 1.19–2.37, *P* ═ 0.004) In participants with current MDD episode, 25(OH)D levels were correlated with IDS score (beta [SE] ═ −0.19 (0.07), *P* ═ 0.003) but not with duration of depression or age of onset (beta [SE] ═ 3.90 (2.69), *P* ═ 0.15 and beta [SE] ═ 0.09 (0.08), *P* ═ 0.22, respectively) Adequate: >75 nmol/L (>30 ng/mL); desirable: 75–50 nmol/L (30–20 ng/mL), insufficient: <50 nmol /L (<20 ng/mL); deficient: <25 nmol/L(<10 ng/mL)
Grudet et al. (2022) [66]	Sweden	GEN-DS study, case-control	243 participants (202 patients with difficult-to-treat depression and 41 healthy controls) Participants with difficult-to-treat depression** had MDD single episode, MDD recurrent episode, chronic MDD or dysthymia	DSM-IV-TR (MINI 6.0, SCID-II) for patients MINI 6.0 for healthy controls LC-MS/-MS	Difficult-to-treat depressed patients had *↓*odds of having increased 25(OH)D levels compared with healthy controls (OR ═ 0.828, *P* ═ 0.02)

IDS: Inventory for Depressive Symptoms; DSM: Diagnostic and Statistical Manual of Mental Disorders; LC-MS-MS: Liquid chromatography-tandem mass spectrometry; BDI: Beck Depression Inventory.

### Cross-sectional studies

We identified 46 eligible cross-sectional studies published between 2008 and 2022, using different techniques for detecting vitamin D, such as: chemiluminescence immunoassay (CIA)—18 studies, radioimmunoassay (RIA)—eight studies, liquid chromatography-tandem mass spectrometry (LC-MS/MS)—10 studies, competitive protein binding assay (CPBA)—four studies, and enzyme immunoassay (EIA)—two studies. Lastly, two of them did not mention the method used for vitamin D detection. For assessing depression levels, 11 out of the 43 studies used the CES-D, nine used the Patient Health Questionnaire-9 (PHQ-9), seven used the GDS, seven used the BDI, two used the Hamilton Rating Scale for Depression (HAMD), three used the Depression Anxiety Stress Scale (DASS-21), and lastly, six used DSM or ICD criteria for depressive disorder. The main findings can be found in Table 4.

#### CES-D

Kim et al. (2020) included only Korean male subjects from routine health check-ups supervised by the National Health Insurance Service and measured their vitamin D levels. The authors’ results indicated a significant association between vitamin D insufficiency and symptoms of depression (*P* ═ 0.002, OR ═ 1.49, 95% CI ═ 1.12–2.00) [67]. In comparison, another study based on health check-ups included only female Korean participants, and according to the regression analysis, vitamin D deficiency had a significant correlation with symptoms of depression (OR ═ 1.55, 95% CI ═ 1.15–2.07) [68]. Mizoue et al. (2014) included data from the Furukawa Nutrition and Health Study. They evaluated both male and female participants, and their levels of circulating vitamin D were inversely associated with depressive symptoms for participants in the highest vitamin D category (OR ═ 0.70, 95% CI ═ 0.43–1.14) [69]. Another health survey contributed vitamin D data for Nanri et al. (2009). However, the authors conducted the study in both November and July to analyze seasonal variation. In the former case, the prevalence of symptoms decreased with higher vitamin D levels (OR for highest vs lowest quartile ═ 0.40, 95% CI ═ 0.16–1.03) [70].

Hoang et al. (2011) described that higher vitamin levels were associated with a decreased risk of depression (OR ═ 0.92, 95% CI ═ 0.87–0.97). This correlation was also observed from October to March (OR ═ 0.87, 95% CI ═ 0.80–0.95), as compared to April–September (OR ═ 0.96, 95% CI ═ 0.89–1.02) [71]. A study focusing on older participants (>50 years old) reported a significant association between low serum vitamin D levels (<30 nmol/L) and depressive symptoms. Di Gessa et al. (2021) investigated data from the English Longitudinal Study of Ageing (ELSA), with subjects aged 50 and older. Only those with insufficient levels of vitamin D throughout the study reported elevated depressive symptoms (OR ═ 1.39, 95% CI ═ 1.00–1.93) [72]. Hoogendijk et al. (2008) included participants from the Longitudinal Aging Study Amsterdam (>65 years old), and their results indicated that vitamin D levels were 14% lower in participants with minor and major depression (*P* < 0.001). Additionally, the severity of depression was associated with low serum vitamin D (*P* < 0.001) [73]. The association was also studied by de Oliveira et al. (2017) using evidence from the ELSA, involving participants older than 50 years. A significant association was found between elevated depressive symptoms and low vitamin D levels (OR ═ 1.58, 95% CI ═ 1.20–2.07 for the lower quartile; OR ═ 1.45, 95% CI ═ 1.15–1.83 for vitamin D level < 30 mmol/L; OR ═ 1.34, 95%CI ═ 1.10–1.62 for <50 mmol/L) [74]. Conversely, other studies could not find an association between the two in the elderly population. Brower-Brolsma et al. (2013) explored the association with cognitive executive function using data from a Dutch population of frail or prefrail elderly participants (>65 years old) from the ProMuscle Study. Although there was no association with depressive symptoms, the results suggested that serum vitamin D was associated with executive functioning (β ═ 0.007, *P* ═ 0.01) and information processing speed (β ═ 0.007, *P* ═ 0.06) [75]. Pan et al. (2009) reported results from the Nutrition and Health of Aging Population in China (NHAPC), where depressive symptoms were less frequent in the highest vitamin D tertile compared to the lowest (OR ═ 0.62, 95% CI ═ 0.46–0.83, *P* ═ 0.002) [76]. Shin et al. (2016) also used data from the Kanbuk Samsung Health Study and reported that the OR for depressive symptoms was increased in patients with vitamin D deficiency (OR ═ 1.158, 95% CI ═ 1.003–1.336, *P* ═ 0.046) [77].

#### PHQ-9

Using data obtained by the Study of Health in Pomerania (SHIP) from northwest Germany, Goltz et al. (2018) found an inverse association between vitamin D levels and depression (OR ═ 0.966, 95% CI ═ 0.951–0.981) from the logistic regression models [78]. Multiple studies used data from the US cross-sectional survey—National Health and Nutrition Examination Survey (NHANES). The results of Zhao et al. (2010) suggested that although the unadjusted odds ratio for moderate to severe depression (OR ═ 0.51, 95% CI ═ 0.31–0.85) and severe depression (OR ═ 0.23, 95% CI ═ 0.06–0.79) was lower in the highest quartile of serum vitamin D, the relationship was not significant after adjusting for lifestyle factors [79]. Moreover, Huang et al. (2018), using data from the same NHANES database (2005–2006), reported similar results, indicating no association between vitamin D concentration and depression (second quartile: OR ═ 0.89, 95% CI ═ 0.58–1.36; third quartile: OR ═ 0.78, 95% CI ═ 0.48–1.26; fourth quartile: OR ═ 0.58, 95% CI ═ 0.36–0.95) [80]. In comparison, another study using data from 2007–2011 and 2013–2014 suggested that participants with vitamin D deficiency had increased odds (by 54%) of reporting symptoms of depression (OR ═ 1.54, 95% CI ═ 1.14–2.07) [81]. One study specifically analyzed this association in female participants from the 2011–2014 survey, but the result was not statistically significant (β ═ 0.001, *P* ═ 0.924) [82]. Rhee et al. (2020) included Korean participants from a national survey conducted in 2014 and tested vitamin D levels in both men and women. The association was statistically significant only in males (IRR ═ 0.74, 95% CI ═ 0.59–0.93). Vitamin D concentration was lower in male participants with moderate depressive symptoms (*P* < 0.001) [83]. During the pandemic—a period characterized by lockdowns, mobility restrictions, and home quarantines, and therefore reduced sun exposure—Anuroj et al. (2022) assessed vitamin D levels in Thai medical students. However, there was no association between serum vitamin D levels and depression (β ═ −0.02, *P* ═ 0.46) [84]. Rabenberg et al. (2016) also considered seasonal variation using nationwide data from the German Health Interview and Examination Survey. Depressive symptoms and vitamin D were inversely associated in the summertime (β ═ −1.03, 95% CI ═ −1.61 to −0.44), but not in the wintertime (β ═ −0.45, 95% CI ═ −0.81 to 0.09) [85]. Based on a cohort from the UK Biobank, a large prospective study involving genotyping, Zhang et al. (2021) reported a significant association between blood levels of vitamin D and depression status (β ═ −0.062, SE ═ 0.003, *P* ═ 5.95 × 10^−96^) [86].

#### GDS

An older study from 2005 (the Health Survey for England–HSE) provided data for Stewart et al. (2010) for a dose-response association investigated in a linear model for depression and vitamin D. Their results were found to be strongly significant, β ═ −1.94, 95% CI ═ −2.67 to −1.20 [87]. The population-based EpiFloripa Aging Study investigated health determinants in Southern Brazil in adults over 60 years old. Ceolin et al. (2020) included the data in their analysis and reported that 15% of participants had depressive symptoms, with a higher prevalence among those with deficient vitamin D levels (OR ═ 23.2, 95% CI ═ 15.6–32.9) and insufficient levels (OR ═ 17.2, 95% CI ═ 11.0–25.9). Moreover, vitamin D-deficient patients had 3.08 times higher odds of depressive symptoms compared to those with sufficient levels (OR ═ 2.27, 95% CI ═ 1.05–4.94) [88]. Furthermore, Albolushi et al. (2022) conducted a cross-sectional study with data from seven healthcare centers in Kuwait from 2020–2021 and reported that low vitamin D levels were a predictor of depressive symptoms. Patients with vitamin D deficiency (OR ═ 19.7, 95% CI ═ 5.60–74.86) and insufficiency (OR ═ 6.40, 95% CI ═ 2.20–19.91) had higher odds of depressive symptoms [89]. Yao et al. (2018) obtained results from the China HAINAN Centenarian Cohort Study (CHCCS), conducted between 2014–2016, and found that low vitamin D was independently a risk factor for depression (OR ═ 1.47, 95% CI ═ 1.08–2.00) [90]. Another study from Europe—the Survey in Europe on Nutrition and the Elderly, a Concerted Action (SENECA)—included both male and female participants aged 70–75 years. Conducted by Brouwer-Brolsma et al. (2012), it showed that although participants with intermediate-to-high levels of serum vitamin D tended to have lower depression scores, these findings were not significant after adjusting for lifestyle factors and demographics (RR ═ 0.73, 95% CI ═ 0.51–1.04; and RR ═ 0.76, 95% CI ═ 0.52–1.11) [91]. Imai et al. (2015) published data from the Age, Gene/Environment Susceptibility–Reykjavik (AGES-Reykjavik) cross-sectional analysis involving older adults living in northern latitudes, and described an increased risk of MDD in men with vitamin D deficiency (OR ═ 2.51, 95% CI ═ 1.03–6.13) [92]. Nonetheless, other authors using data from the Korean Urban Rural Elderly (KURE) study, which included participants older than 65, reported a similar significant association only in male participants, with ORs of 1.74 (95% CI ═ 0.85–3.58), 2.50 (95% CI ═ 1.20–5.18), and 2.81 (95% CI ═ 1.15–6.83) for the following vitamin D concentrations: 20–29.9, 10–19.9, and <10 ng/mL, respectively [93].

#### BDI

Chen et al. (2020) tested the hypothesis that life stress and mental health are associated with vitamin D in young adults, taking into consideration racial disparities. After adjustment for gender, race, and age, the results suggested that higher vitamin D serum levels were related to lower scores on the BDI (β ═ −0.16, *P* ═ 0.018). Only white participants’ serum concentrations were related to BDI scores (*P* < 0.05) [94]. Another study from Nepal, with participants older than 18, identified a significant association between gender, residence, religion, marital status, and vitamin D deficiency with significant clinical depression (OR ═ 4.4, 95% CI ═ 1.4–12.9, *P* ═ 0.007) [95]. Jääskeläinen et al. (2014) conducted their research using a Finnish study from 2000–2001, based on a representative sample of participants aged 30–79 years. Participants with high vitamin D levels had a lower risk of depression (OR ═ 0.65, 95% CI ═ 0.46–0.93) [96]. Outdoor activities and vitamin D supplementation were studied as modifiable lifestyle components for depressive symptoms in office workers in the research by Jin et al. (2016). For the group of participants with sufficient levels of vitamin D, the odds of having symptoms of depression were lower (OR ═ 0.121 in men, OR ═ 0.114 in women) [97]. Another study by Bouloukaki et al. (2022) investigated the seasonal impact on vitamin D levels in Crete, Greece, in a primary care setting. Depressive symptoms had an independent association with vitamin D deficiency (OR ═ 3.769, 95% CI ═ 0.984–14.443, *P* ═ 0.04) [98]. Lee et al. (2011) included in his study data from the European Male Ageing Study (EMAS), a prospective cohort study of males aged 40–79 years, and assessed the relationship between vitamin D and depression, revealing an inverse association between the two (OR ═ 1.74, 95% CI ═ 1.00–3.00) [99]. Terock et al. (2020) studied vitamin D levels in the general population from the SHIP-1 and their association with both lifetime and current depressive symptoms. However, the results indicated there was no association between the two (OR ═ 1.004, 95% CI ═ 0.753–1.339, *P* ═ 0.977; β ═ −0.753, SE ═ 0.438, *P* ═ 0.086) [100].

#### DASS-21

Only three cross-sectional studies used this scale for measuring depressive symptoms; however, all of them reported similar results regarding the association between vitamin D and symptoms of depression. Additionally, all of them were conducted in countries with increased sunlight exposure [101–103]. Black et al. (2014) investigated this association in adult participants from Australia, and the results suggested that in males, higher vitamin D levels were associated with reduced depressive symptoms. Specifically, an increase in vitamin D levels of 10 nmol/L corresponded to a 9% decrease in the DASS score (RR ═ 0.91, 95% CI ═ 0.87–0.95, *P* < 0.001) [101]. Female participants from a tropical country (Malaysia) were found to have low levels of vitamin D and abnormal depression scores. Furthermore, participants at risk of depression were more likely to have low vitamin D levels (aOR ═ 1.88, 95% CI ═ 1.27–2.79) [102]. Another study, which took into consideration sunlight exposure, included adult Jordanian participants. The results of logistic regression indicated that the odds ratio for depression was higher in adults with lower levels of vitamin D (<30 ng/mL) (OR ═ 1.32). Moreover, the odds ratio decreased linearly with higher quartiles of vitamin D [103].

#### HAMD

Some of the included studies used the score in patients already diagnosed with MDD [90, 91]. Results suggested that serum vitamin D (β–0.154, t–1.043, P0.303) was not a predictor of HAMD score [104]. Furthermore, patients with depression and hypovitaminosis were prone to cognitive impairment (β ═ −0.288, 0.028) [105].

#### Diagnostic and statistical manual for mental disorders criteria or ICDs criteria for depressive disorder

Schaad et al. (2019) evaluated the impact of vitamin D in a group of individuals serving army duty in the USA and found a significant association with depression among participants with vitamin D deficiency compared to those with normal levels, OR ═ 1.22, 95% CI ═ 1.11–1.33, *P* < 0.001 [106]. Another study published in 2019 used the UK Biobank and explored the association between two subtypes of depression (treatment-resistant and atypical depression). The results suggest that an increase in vitamin D was associated only with a reduced risk of atypical depression when comparing patients with atypical depression to probable MDD patients or the general population [107]. Vidgren et al. (2017) included in their research data from the Kuopio Ischaemic Heart Disease Risk Factor Study (KIHD), a population-based study of cardiovascular diseases in middle-aged and older individuals from Finland. The results indicate that low vitamin D levels were associated with a higher risk of depression, OR ═ 1.64, 95% CI ═ 1.03–2.59; however, seasonal variation had no impact on the odds of depression, OR ═ 1.12, 95% CI ═ 0.79–1.60 [108]. Köhnke et al. (2020) conducted an analysis using the BiDirect study, which included two cohorts: one with individuals already diagnosed with MDD and a control group from the population of Münster. Participants with mild depression had the highest odds of vitamin D deficiency, OR ═ 2.69, 95% CI ═ 1.75–4.14, particularly those with the atypical subtype, OR ═ 3.58, 95% CI ═ 1.45–8.83 [109]. Some of these studies evaluated older populations, in which lower levels of vitamin D were associated with depression [110, 111]. Voshaar et al. (2014) used data from the Netherlands Study of Depression in Older People and found that lower levels of vitamin D were associated with depression, Cohen’s *d* ═ 0.28, 95% CI ═ 0.07–0.49, *P* ═ 0.033 [111]. Similar results were reported by Lapid et al. (2018), where participants with severe vitamin D deficiency were twice as likely to have depression, OR ═ 2.093, 95% CI ═ 1.092–4.011, *P* ═ 0.026 [110]. Lastly, a study from Northern Norway with adult participants by Kjaergaard et al. (2011) presented the results separately for smokers and non-smokers, concluding that hypovitaminosis could be used to predict depression in female participants among both smokers (*P* ═ 0.015) and non-smokers (*P* ═ 0.003) [112].

## Discussion

Traditionally recognized for its role in calcium homeostasis, vitamin D has also been implicated in non-skeletal processes, such as modulating immunity and regulating metabolic pathways. These various roles provide a plausible explanation for the association between vitamin D and mental health outcomes, in this case—depression [113, 114]. In the present systematic review, we have investigated the association between vitamin D and depressive symptoms. We included in our research studies with longitudinal designs [47–56], cross-sectional and longitudinal designs [57–61, 63], case-control [64–66], and, lastly, cross-sectional designs [67–112]. Throughout the included manuscripts, vitamin D levels were measured using six different techniques: CIA [55, 58, 67, 68, 71, 72, 74, 77, 78, 85–88, 92, 93, 95, 99, 100, 103, 104, 107, 112], RIA [54, 59–63, 76, 79, 83, 90, 96–98, 102], LC-M/SM [47–49, 52–54, 65, 66, 75, 80–82, 89, 101, 105, 108–111], CPBA [50, 69, 70, 73, 91], EIA [64, 84, 94], and ECLIA [51, 57]. Depressive symptoms were assessed using eight different questionnaires: IDS-30SR [47, 52–55], CES-D [48–51, 56, 57, 59–61, 67–77], PHQ [58, 78–86], GDS [62, 63, 87–93], BDI [64, 94–100], DASS-21 [101–103], HAMD [104, 105], and MADRS [66].

Currently, classification of vitamin D levels remains an ongoing controversy. A relatively recent expert consensus paper states that a 25(OH)D level <20 ng/mL (<50 nmol/L) indicates vitamin D deficiency; 20 ng/mL (50 nmol/L)–30 ng/mL (75 nmol/L) is considered insufficiency, while a concentration of 30–50 ng/mL (75–125 nmol/L) is sufficient [115]. Unfortunately, there is no consensus on 25(OH)D levels for patients with depression. Although speculative, the evidence summarized in this review suggests that a 25(OH)D level ≤30 nmol/L, indicative of vitamin D deficiency, may be linked to a higher likelihood of experiencing significant depressive symptoms.

The literature search was completed in April 2023. After interpreting our results and drawing conclusions, we conducted a subsequent review of the literature to identify any studies published after this date that could potentially affect our conclusions. Up to 2025, we did not find any major studies that could materially affect our review; however, the findings of studies published after this date may impact the results and conclusions of the current investigation. We started by analyzing the findings from longitudinal studies, which exhibited inconsistencies due to differences in sample populations, study designs, or the methods used to assess both depression and vitamin D levels. This variability suggests that the role of vitamin D in depression may be just one of multiple contributing factors. Furthermore, the conflicting results imply that the relationship between vitamin D and depression might be affected by additional lifestyle factors that fluctuate over time. We then proceeded to investigate studies with both longitudinal and cross-sectional designs. Longitudinal studies have generally demonstrated a minimal to no association between vitamin D deficiency and depression. In contrast, most cross-sectional studies suggest the opposite, indicating that vitamin D deficiency is linked to an increased risk of depressive symptoms; however, these findings do not provide conclusive evidence about the long-term progression of the disorder.

The results from the case-control studies focused on the association with MDD [64–66], suggesting that the deficiency could be linked to the occurrence of the disorder. Moreover, higher vitamin D levels were associated with a greater treatment response, suggesting that vitamin supplementation could increase the effectiveness of depression treatment in resistant cases [66]. Further studies are needed to determine whether vitamin D plays a role in the course and prognosis of clinically diagnosed depression over time. Additionally, gender is an important factor that influences circulating vitamin D levels and their relationship with depressive symptoms. Most of the cross-sectional studies that included both male and female participants with low levels of circulating vitamin D concluded that they had an increased likelihood of depressive symptoms, or that lower levels of vitamin D were found in participants with a prior history of depression. However, the studies differed in whether they reported gender-specific associations [67–74, 76–78, 81, 83, 85–90, 92–99, 101–103, 106–112]. Some of the included studies that focused their research on male subjects only reported a significant association between vitamin D insufficiency and symptoms of depression [67], or that higher vitamin D levels were associated with reduced depressive symptoms [101]. In contrast, other studies that included only female participants found a significant correlation between vitamin D deficiency and symptoms of depression [68, 82]. Moreover, our results suggested that hypovitaminosis could be used in predicting depression and anxiety [112]. The majority of studies reported a statistically significant association between depression and vitamin D [47, 49–52, 54, 56–58, 62, 64–74, 76–78, 81, 83, 85–90, 92–99, 101–103, 106–112], although several studies did not find any measurable association [75, 80, 84, 91, 100, 104, 105, 107]. This could be explained by the lack of data on confounding factors, such as sun exposure, latitude, skin pigmentation, use of sunblock, skin-covering clothes, or dietary intake among the included participants. When taking into consideration working hours, shift work has been significantly associated with poor sleep quality, which leads to depressive symptoms. Moreover, latitude also strongly influences vitamin D levels by affecting the amount of radiation that reaches the Earth’s surface (e.g., higher latitudes receive less direct sunlight, especially in fall and winter, thus negatively influencing vitamin D synthesis). Another important covariate is the patient’s comorbidities, which can lead to reduced outdoor activity, metabolic changes that impair the conversion of vitamin D to its active form, inflammation, medications that reduce vitamin D levels over time (e.g., corticosteroids), or gastrointestinal disorders that impair vitamin absorption [116]. Considering seasonal variation, depressive symptoms and vitamin D were inversely associated in the summertime but not in the wintertime [85]. Moreover, during the Coronavirus pandemic lockdowns and quarantines, outdoor activity was limited, and people were confined to their homes, which in turn reduced sunlight exposure and led to a decline in vitamin D levels. Another challenge during this period was the shortage of food supplies due to lockdowns, transportation issues, restrictions, and labor shortages, making it difficult to ensure a steady supply. Several studies examining vitamin D levels in older adults have found that deficiency is a prevalent issue within this group [72–74, 87–91, 108, 110, 111]. As individuals age, their skin becomes less efficient at producing vitamin D. Additionally, older adults often experience reduced sun exposure due to mobility limitations or living in care facilities, where outdoor activities are limited. Other potential factors contributing to lower vitamin D levels in this population include dietary insufficiencies, chronic illnesses, medication use, and the natural decline in renal function associated with aging.

**Table 4:** Main findings of the cross-sectional studies

**Author and year**	**Study location**	**Study design**	**Study population**	**Depression assessment/Vitamin D detection method**	**Main findings**
Kim et al., 2020 [67].	Korea	Cross-sectional	Male adults undergoing health check-up-NHIS	CES-D; ≥16 depression; CIA	A significant association between vitamin D insufficiency and symptoms of depression (*P* ═ 0.002 OR ═ 1.49, 95% CI ═ 1.12 –2.00)
Rhee et al., 2014 [83]	Korea	Cross-sectional	General population from Korea National Health and Nutrition Examination Survey 2014	PHQ-9 ≥10–depression; RIA	The association was statistically significant only in males (IRR ═ 0.74, 95% CI ═ 0.59–0.93)
Chen et al., 2020 [94]	USA	Cross-sectional	General population	Beck depression inventory; EIA	The results suggested that higher vitamin D serum levels were related to lower scores on BDI (β ═ −0.16, *P* ═ 0.018) Only white participants’ serum concentration were related to BDI scores (*P* < 0.05)
Bigman et al., 2022 [81]	USA	Cross-sectional	General population from NHANES 2007–2010 and 2013–2014	PHQ-9-a score ≥5–depression symptoms; LC-MS-MS	The results suggested that participants with vitamin D deficiency had increased odds (54%) to report symptoms of depression (OR ═ 1.54, 95% CI ═ 1.14–2.07)
Zhu et al., 2019 [104]	China	Cross-sectional	Patients diagnosed with depression	ICD-10, Hamilton depression Rating Scale-24 items; CIA	Results suggested that serum vitamin D (β ═ −0.328, t ═ −2322, *P* ═ 0.025, R2 ═ 0.111) was a predictor of HAMD score
de Oliveira et al., 2018 [74]	United Kingdom	Cross-sectional	Older population (>50 years old) (ELSA)	CES-D-8, depression ═ ≥4; CIA	The association was significant between elevated depressive symptoms and low vitamin D levels (O ═ 1.58, 95% CI ═ 1.20–2.07 lower quartile, OR ═ 1.45, 95% CI ═ 1.15–1.83 for vitamin D level <30 mmol/L, OR ═ 1.34, 95% CI ═ 1.10–1.62 for <50 mmol/L)
Sherchand et al., 2018 [95]	Nepal	Cross-sectional	General population (over 18 years old)	BDI, BDI ≥ 20 ═ clinical depression; CIA	Significant association between gender, residence, religion, marital status, and vitamin D deficiency with significant clinical depression (OR ═ 4.4, 95% CI ═ 1.4–12.9, *P* ═ 0.007)
Goltz et al., 2017 [78]	Germany	Cross-sectional	General population (SHIP)	PHQ-9, None < 5, mild 5–9, Moderate 10–14 moderate–severe >═ 15; CIA	An inverse association between vitamin D levels and depression (OR ═ 0.966, 95% CI ═ 0.951–0.981)
Huang et al., 2018 [80]	USA	Cross-sectional	General population from NAHNES 2005–2006	PHQ-9-a score of ≥10–depression; LC-MS/-MS	No association between vitamin D concentration and depression (OR for the second quartile ═ 0.89, 95% CI ═ 0.58–1.36; OR for the third quartile ═ 0.78, 95% CI ═ 0.48–1.26; OR for the fourth quartile ═ 0.58, 95% CI ═ 0.36–0.95)
Belzeaux et al., 2018 [105]	Canada	Cross-sectional	Patients between 18–65 years old diagnosed with depression	DSM-IV, Hamilton depression rating scale-21 item (HDRS-21); LC-MS-MS	Results suggested that serum vitamin D (β ═ −0.328, t ═ −2322, *P* ═ 0.025, R2 ═ 0.111) was a predictor of HAMD score
Vidgren et al., 2018 [108]	Finland	Cross-sectional	General population (middle age or older) from the Kuopio Ischaemic Heart Disease Risk factor study	DSM-III scale, DSM-III scale score ≥4 or undergoing current antidepressant therapy ═ depression diagnosis; LC-MS-MS	The results indicate that low vitamin levels were associated with higher rick of depression (OR ═ 1.64, 95% CI ═ 1.03–2.59), however, seasonal variation had no impact on depression odds (OR ═ 1.12, 95% CI ═ 0.79–1.60)
Hoogendijk et al., 2008 [73]	The Netherlands	Cross-sectional	Older population (55–85 years old) (Longitudinal Aging study amsterdam)	CES-D score ≥16 ═ depression; CPBA	Vitamin D levels were 14% lower in participants with minor depression and major depression (*P* < 0.001), also, the severity of depression was associated with low serum vitamin D (*P* < 0.001)
Jääskeläinen et al., 2015 [96]	Finland	Cross-sectional	General population (30–79 years old) from the Health 2000 Survey	Beck Depression Inventory, depression ═≥ 10 points; Munich-composite International diagnostic Interview for the diagnosis of depressive disorder and major depressive disorder; RIA	High vitamin D level participants had a lower risk of depression (OR ═ 0.65, 95% CI ═ 0.46–0.93)
Mizoue et al., 2015 [69]	Japan	Cross-sectional	General population from Furukawa Nutrition and Health Study	CES-D score ≥16 ═ depression; CPBA	The levels of circulating vitamin D were inversely associated with depressive symptoms for participants with the highest category of vitamin D levels OR ═ 0.70, 95% CI ═ 0.43–1.14
Pan et al., 2009 [76]	China	Cross-sectional	Middle aged and elderly population (50–70 years old)	CES-D score ≥16 ═ depression; RIA	Depressive symptoms were less frequent in the top vitamin D tertile compared to the lowest (OR ═ 0.62, 95% CI ═ 0.46-0.83, *P* ═ 0.002)
Moy et al., 2017 [103]	Malaysia	cross-sectional	Women	Depression, Anxiety and Stress Scale (DASS), depression score >9 ═ risk of depression; CIA	Female participants were found to have low levels of vitamin D and abnormal depression scores. Furthermore, participants at risk of depression were associated with low vitamin D levels (aOR ═ 1.88, 95% CI ═ 1.27–2.79)
Brouwer-Brolsma et al., 2012 [91]	Several European countries (Belgium, Denmark, France, Hungary, The Netherlands, Switzerland, Greece, Spain, Portugal)	Cross-sectional	Older population (70–75 years old) from the SENECA study	Geriatric depression scale; CPBA	Participants that had intermediate-high levels of serum vitamin D had a tendency for a lower depression score. When taking into consideration lifestyle factors and demographics these finding were not significant (RR ═ 0.73, 95% CI ═ 0.51–1.04 and RR ═ 0.76, 95% CI ═ 0.52–1.11)
Zhao et al., 2010 [79]	USA	Cross-sectional	General population from NHANES 2005–2006	PHQ-9 score≥10 moderate-severe depression; RIA	Even though the unadjusted Odds ratio for moderate-severe depression (OR ═ 0.51, 95% CI ═ 0.31–0.85) or severe depression (OR ═ 0.23, 95% CI ═ 0.06–0.79) was lower in the highest quartile of serum vitamin D, the relationship between the two was not significant when adjusting lifestyle factors
Nanri et al., 2009 [70]	Japan	Cross-sectional	General population (employees of two municipal offices)	CES-D score ≥16 ═ depression; CPBA	In November the symptom prevalence decreased with vitamin D levels; OR for the highest –owest quartile ═ 0.40, 95% CI ═ 0.16–1.03)
Song et al., 2016 [93]	South Korea	Cross-sectional	Older population (older than 65) from the Korean Urban Rural Elderly study	Geriatric depression Scale -Short Form, GDS-SF ≥ 6═ depression; CIA	Significant association only in male participants with OR=1,74 (0.85-3.58), OR ═ 2.50 (1.20–5.18), OR ═ 2.81 (1.15–6.83) for the following vitamin D concentrations: 20–29.9, 10–19.9, <10 ng/mL
Brouwer-Brolsma et al., 2013 [75]	The Netherlands	Cross-sectional	Older population (>65 years old) from SENECA	CES-D 20; LC-MS-MS	Although there was no association between the two, the results suggested that serum vitamin D was associated with executive functioning (b ═ 0.007, *P* ═ 0.01) and information processing speed (b ═ 0,007, *P* ═ 0.06)
Kjærgaard et al., 2011 [112]	Norway	Cross-sectional	General population from The Tromsø study	Hopkins symptoms check list 10 (SCL-10), SCL-10 ≥ 1.85 ═ depression; CIA	For the female participants hypovitaminosis could be used in predicting depression for both smokers (*P* ═ 0.015) and non-smokers (*P* ═ 0.003)
Shin et al., 2016 [77]	South Korea	Cross-sectional	General population	CES-D 20 score ≥21 ═ depression; CIA	The OR for depressive symptoms was increased in vitamin D deficiency patients (OR ═ 1.158, 95% CI ═ 1.003–1.336, *P* ═ 0.046), however, they found no significant association with C Reactive Protein
Ceolin et al., 2020 [88]	Brasil	Cross-sectional	Data from the EpiFloripa aging study–longitudinal study with population older than 60	GDS-15, GDS-15 ≥ 6 ═ depression; CIA	15% of participants had depressive symptoms with a higher prevalence for those with deficient vitamin D levels (23.2, 95% CI ═ 15.6–32.9) and insufficient levels (17.2, 95% CI ═ 11.0–25.9). Moreover, vitamin D deficient patients had 3.08 times higher odds for depressive symptoms in comparison to those with sufficient levels (OR ═ 2.27, 95% CI ═ 1.05–4.94)
Lapid et al., 2013 [110]	USA	Cross-sectional	Older population (≥60 years old)	ICD-8; LC-MS-MS	Severe vitamin deficiency participants were twice as probable to have depression (OR ═ 2.093, 95% CI ═ 1.092–4.011, *P* ═ 0.026)
Jaddou et al., 2012 [102]	Jordan	Cross-sectional	General population (≥25 years old)	Depression anxiety and stress scale (DASS21)-depression scale, DASS21-D ≥ 14 ═ depression; RIA	The results of logistic regression indicated the OR for depression was higher in adults with lower levels of vitamin D (<30 ng/ml) (OR ═ 1.32). Moreover, the OR decreased linearly with higher quartiles of vitamin D
Hoang et al., 2011 [71]	USA	Cross-sectional	General population (CCLS)	CES-D score ≥ 10 ═ depression; CIA	Higher vitamin levels were associated with decreased risk of depression (OR ═ 0.92, 95% CI ═ 0.87–0.97). Also, this correlation was present from October to March OR ═ 0.87, 95% CI ═ 0.80–0.95 (as compared to April-September OR ═ 0.96, 95% CI ═ 0.89–1.02)
Stewart et al., 2010 [87]	United Kingdom	Cross-sectional	Older population from health survey for England (≥65 years old)	GDS10, GDS10 ≥ 3 ═ depression; CIA	Their results were found to be strongly significant (β ═ −1.94, 95% CI ═ −2.67,−1.20)
Albolushi et al., 2022 [89]	Kuwait	Cross-sectional	Older population (>65 years old)	GDS-15, GDS-15 ≥ 5 ═ depression; LC-MS-MS	Low vitamin D level was a predictor for depressive symptoms. Vitamin D deficiency (OR ═ 19.7, 95% CI ═ 5.60–74.86) and vitamin D insufficiency (OR ═ 6.40, 95% CI ═ 2.20–19.91) patients had higher odds for depressive symptoms
Rabenberg et al., 2016 [85]	Germany	Cross-sectional	General population from the German health inteview and examination survey for Adults 2008–2011	PHQ-9, depression ═ PHQ-9 ≥ 10; CIA	Depressive symptoms and vitamin D were inversely associated in the summertime (β ═ −1.03, 95% CI ═ −1.61 and 0.44), but not in wintertime (β ═ −0.45, 95% CI ═ −0.81 and 0.09)
Di Gessa et al., 2021 [72]	United Kingdom	Cross-sectional	Older population (>50 years old) (ELSA)	CES-D8 score ≥ 4 ═ depression; CIA	Only those with insufficient levels of vitamin D throughout the study reported elevated depressive symptoms (OR ═ 1.39, 95% CI ═ 1.00–1.93)
Zhang et al., 2021 [86]	United Kingdom	Cross-sectional	General population from the UK Biobank (age 40–69)	PHQ-9; CIA	Significant association between blood levels of vitamin D and depression status (β ═ −0.062, SE ═ 0.003, *P* ═ 5.95 x 10–96)
Anuroj et al., 2022 [84]	Thailand	Cross-sectional	Medical students of year 4–5	PHQ 9-A-depression ═ PHQ-9 ≥ 10; EIA	There was no association between serum vitamin levels and depression (β ═ −0.02, *P* ═ 0.46)
Imai et al., 2015 [92]	Iceland	Cross-sectional	Older population (66–96 years old) from AGES-Reykjavik	Geriatric Depression Scale-15(GDS-15), the patients completed MINI (DMS-IV criteria) if GDS-15 score ≥6, reported anxiety symptoms/history of antidepressant treatment/received a diagnosis of depression from a doctor/were taking antidepressant medication at the moment of the study; CIA	Increased risk of having major depressive disorder for men with deficient vitamin D (OR ═ 2.51, 95% CI ═ 1.03–6.13)
Black et al., 2014 [101]	Australia	Cross-sectional	General population (children of participants to the Western Australian pregnancy cohort study	Depression, Anxiety and Stress Scale 21-item (DASS-21); LC-MS-MS	In males, higher vitamin D levels were associated with reduced depressive symptoms. More specifically, an increase in vitamin D levels of 10 nmol/L corresponded to a decrease in DASS score by 9% (RR ═ 0.91, 95% CI ═ 0.87–0.95, *P* < 0.001)
Yao et al., 2018 [90]	China	Cross-sectional	Longevous Population (over 100 years old)	GDS-15, GDS-15 ≥ 6 ═ depression; RIA	Low vitamin D was independently a risk factor for depression (OR ═ 1.47, 95% CI ═ 1.08–2.00)
Voshaar et al., 2014 [111]	The Netherlands	Cross-sectional	MDD patients (60–93 years old) from The Netherlands study of depression in older People	DSM-IV-R, Inventory of depressive symptomatology; LC-MS-MS	Lower levels of vitamin D were found in participants with depression (Cohen’s d ═ 0.28, 95% CI ═ 0.07–0.49, *P* ═ 0.033)
Jin et al., 2017 [97]	South Korea	Cross-sectional	General population (≥30 years old)	Beck Depression Inventory II (BDI-II), BDI-II ≥ 10 ═ depression; no depression group ═ BDI < 10; mild depression group ═ 10 ≤ BDI ≤ 15; moderate depression group 16 ≤ BDI ≤ 23, severe depression >23; RIA	The association was statistically significant only in males (IRR ═ 0.74, 95% CI ═ 0.59–0.93). Vitamin D concentration was lower for male participants with moderate depressive symptoms (*P* < 0.001)
Kwon et al., 2015 [68]	South Korea	cross-sectional	Indoor female workers	CES-D score ≥ 16 ═ depression; CIA	Vitamin D deficiency had a significant correlation with symptoms of depression (OR ═ 1.55, 95% CI ═ 1.15–2.07)
Bouloukaki et al., 2022 [98]	Greece	cross-sectional	General population (≥18 years old) doing a health check-up between 2015 and 2018	Beck depression inventor II > 10 depressive symptoms, RIA	Depressive symptoms had an independent association with vitamin D deficiency (OR ═ 3.769, 95% CI ═ 0.984–14.443, *P* ═ 0.04)
Li et al., 2022 [82]	USA	cross-sectional	Female participants in the National Health and Nutrition Examination Survey 2011–2014 (NHANES)	PHQ-9 score ≥ 5 ═ depression; LC-MS-MS	The association between vitamin D and symptoms of depression was not statistically significant (β ═ 0.001, *P* ═ 0.924)
Terock et al., 2020 [100]	Germany	Cross-sectional	General population	Lifetime depressive symptoms were assessed with CID-S and current depressive symptoms with BDI II; CIA	The results indicated there was no association between lifetime depressive symptoms/ current depressive symptoms and levels of vitamin D (OR ═ 1.004, 95% CI ═ 0.753–1.339, *P* ═ 0.977; β ═ −0.753, SE ═ 0.438, *P* ═ 0.086)
Lee et al., 2011 [99]	Florence, Leuven, Manchester, Santiago de Compostela, Szeged, Tartu, Łódź, Malmö	Cross-sectional	Non institutionalised men between 40–79 years old	BDI II ≥14-depression; CIA	The authors assessed the relationship between vitamin D and depression and the results revealed an inverse association between the two (OR ═ 1.74, 95% CI ═ 1.00–3.00)
Köhnke et al., 2020 [109]	Germany	Cross-sectional	General population (35–65 years old)	DSM-IV criteria; LC-MS-MS	Participants with mild depression had the highest odds of vitamin D deficiency (OR ═ 2.69, 95% CI ═ 1.75–4.14), specifically, the atypical subtype (OR ═ 3.58, 95% CI ═ 1.45–8.83)
Schaad et al., 2019 [106]	USA (Watertown, New York; Fairbanks, Alaska; Killeen, Texas; Tacoma, Washington; El Paso, Texas; and Fayetteville, North Carolina)	Cross-sectional	General population (individuals serving army duty; 18–64 years old)	DSM–V criteria; Method of vitamin D detection not specified	Participants with vitamin D deficiency had a significant association with depression compared to the ones with normal levels (OR ═ 1.22, 95% CI ═ 1.11–1.33, *P* < 0.001)
Arathimos et al., 2021 [107]	United Kingdom	Cross-sectional	General population from the UK Biobank (age 40–69)	TRD: ≥2 diagnostic codes for unipolar depression and ≥2 switches between antidepressant drugs AD: DSM-5 criteria for probable lifetime MDD based on CIDI Short Form and reported hypersomnia and weight gain; CIA	Increased serum vitamin D was associated with a decreased risk of AD compared to probable MDD controls (patients meeting criteria for probable lifetime MDD but did not meet criteria for AD) (OR ═ 0.927, 95%CI ═ 0.882–0.974, *P* ═ 0.002718) or general population (OR ═ 0.917, 95% CI ═ 0.874–0.962, *P* ═ 0.000362)

CIA: Chemiluminescence immunoassay; IDS: Inventory for Depressive Symptoms; DSM: Diagnostic and Statistical Manual of Mental Disorders; LC-MS-MS: Liquid chromatography-tandem mass spectrometry; BDI: Beck Depression Inventory; RIA: Radioimmunoassay; CES-D: Centre for Epidemiological Studies-Depression; ICD: International Classification of Disease; GDS: Geriatric Depression Scale; CPBA: Competitive protein binding assay; ELSA: English Longitudinal Study of Ageing; DASS: Depression, Anxiety and Stress Scale; HAMD: Hamilton Rating Scale for Depression; SENECA: Survey in Europe on Nutrition and the Elderly, a Concerted Action; PHQ: Patient Health Questionnaire-9.

Taking into consideration our inclusion and exclusion criteria, most of the included studies used self-report scales to assess depressive symptoms rather than standardized diagnostic criteria for diagnosing MDD. Thus, establishing a clear connection between vitamin D and clinically diagnosed depression remains insufficiently explored and represents a research gap that deserves further investigation. Another important factor that may interact with the vitamin D–depression association is genetics. For example, emerging evidence shows that VDR polymorphisms (such as CYP27B1 and group-specific component (GC), which encodes vitamin D binding protein) are related to vitamin D metabolism and could influence susceptibility to depressive symptoms [117–118]. These genetic interactions may explain the inconsistent results. However, while low levels of vitamin D have been shown to be implicated in the pathogenesis of neurological conditions (such as multiple sclerosis), the low prevalence of depression in populations with vitamin D deficiency, such as in Asia, supports the hypothesis that both genetic and ethnic backgrounds may influence disease vulnerability [119]. Therefore, future research should explore this interaction to clarify genetic variation and its contribution to the association between vitamin D and depression.

This systematic review has several strengths and limitations that should be acknowledged. We performed a methodologically sound and transparent systematic review of the existing literature using PRISMA guidelines, extracting data from three archives. We identified articles and assessed their eligibility for our comprehensive research, including both longitudinal and cross-sectional studies. Another advantage is that the included manuscripts studied diverse populations from around the world. Moreover, to our knowledge, this is the first systematic review to investigate the association between 25-hydroxyvitamin D serum levels or categories of 25-hydroxyvitamin D levels and clinically diagnosed depression in adults. Some of the included studies reported no association between vitamin D and depression. However, we must highlight that vitamin D detection methods and depression questionnaires varied across the included studies; therefore, the results may differ. Another important limitation of this systematic review is the inability to perform a meta-analysis due to the heterogeneity of the included studies in terms of study design, populations studied, assessment of depression with different scales and cut-offs, vitamin D measurement methods, and the distinction between vitamin D2 and D3. This review also highlighted the limited number of longitudinal studies investigating the relationship between vitamin D and depression over time. Existing longitudinal research often shares certain methodological limitations, such as focusing primarily on older adults (≥65 years), failing to assess participants based on established criteria for MDD, and utilizing only long follow-up periods (e.g., 3–5 years). Future research would benefit from longitudinal studies targeting younger populations and employing shorter follow-up intervals.

## Conclusion

Vitamin D is important to many brain processes (i.e., neuroimmunomodulation, neuroplasticity), which suggests that it also plays a role in mental health. Our systematic review studied its implications in depression. Given the evidence provided by the cross-sectional research examined in this review, an association between the two conditions was observed. However, further longitudinal studies are needed to establish a causal relationship. These findings have significant health implications, particularly considering that vitamin D supplementation for the general population is both cost-effective and generally free of adverse effects.

## Supplemental data

Supplemental data are available at the following link: https://www.bjbms.org/ojs/index.php/bjbms/article/view/12331/3863.
